# The Effect of Working Position on Trunk Posture and Exertion for Routine Nursing Tasks: An Experimental Study

**DOI:** 10.1093/annhyg/met071

**Published:** 2013-12-26

**Authors:** Sonja Freitag, Rachida Seddouki, Madeleine Dulon, Jan Felix Kersten, Tore J. Larsson, Albert Nienhaus

**Affiliations:** ^1.^Department for the Principle of Prevention and Rehabilitation, BGW–Institution for Statutory Accident Insurance and Prevention in the Health and Welfare Services, Pappelallee 33/35/37, 22089 Hamburg, Germany;; ^2.^Institute for Health Services Research in Dermatology and Nursing, UKE–University Medical Centre Hamburg-Eppendorf, Martinistraße 52, 20246 Hamburg, Germany;; ^3.^Institute for Medical Biometry and Epidemiology, UKE–University Medical Centre Hamburg-Eppendorf, Martinistraße 52, 20246 Hamburg, Germany;; ^4.^Centre for Health and Building, KTH–Royal Institute of Technology, Marinens väg 30, 136 40 Haninge, Sweden

**Keywords:** bed height, bending, musculoskeletal disorders, nursing, perceived exertion, trunk posture

## Abstract

**Objectives:**

To examine the influence of the two following factors on the proportion of time that nurses spend in a forward-bending trunk posture: (i) the bed height during basic care activities at the bedside and (ii) the work method during basic care activities in the bathroom. A further aim was to examine the connection between the proportion of time spent in a forward-bending posture and the perceived exertion.

**Methods:**

Twelve nurses in a geriatric nursing home each performed a standardized care routine at the bedside and in the bathroom. The CUELA (German abbreviation for ‘computer-assisted recording and long-term analysis of musculoskeletal loads’) measuring system was used to record all trunk inclinations. Each participant conducted three tests with the bed at different heights (knee height, thigh height, and hip height) and in the bathroom, three tests were performed with different work methods (standing, kneeling, and sitting). After each test, participants rated their perceived exertion on the 15-point Borg scale (6 = no exertion at all and 20 = exhaustion).

**Results:**

If the bed was raised from knee to thigh level, the proportion of time spent in an upright position increased by 8.2% points. However, the effect was not significant (*P* = 0.193). Only when the bed was raised to hip height, there was a significant increase of 19.8% points (reference: thigh level; *P* = 0.003) and 28.0% points (reference: knee height; *P* < 0.001). Bathroom tests: compared with the standing work method, the kneeling and sitting work methods led to a significant increase in the proportion of time spent in an upright posture, by 19.4% points (*P* = 0.003) and 25.7% points (*P* < 0.001), respectively. The greater the proportion of time spent in an upright position, the lower the Borg rating (*P* < 0.001) awarded.

**Conclusions:**

The higher the proportion of time that nursing personnel work in an upright position, the less strenuous they perceive the work to be. Raising the bed to hip height and using a stool in the bathroom significantly increase the proportion of time that nursing personnel work in an upright position. Nursing staff can spend a considerably greater proportion of their time in an ergonomic posture if stools and height-adjustable beds are provided in healthcare institutions.

## INTRODUCTION

Nursing staff suffer a higher frequency of back complaints in the lower back area than workers in other professions (Menzel, 2004; Podniece, 2007). In this context, many studies have examined manual patient transfers and identified them as a risk factor (Engkvist *et al.*, 2000; Byrns *et al.*, 2004). However, patient transfers take up <4% of a work shift (Hodder *et al.*, 2010) and nurses have many other types of work to perform, such as washing patients, making beds, or changing dressings (Harber *et al.*, 1987; Freitag *et al.*, 2007). In a previous study, we found that nurses often bend forward (sagittal trunk inclination) when performing the pre-mentioned activities or work for extended periods in an inclined posture (static trunk inclination) (Freitag *et al.*, 2007). These trunk postures are under discussion as additional risk factors for the development of back complaints (National Institute for Occupational Safety and Health, 1997; Hodder *et al.*, 2010; Habibi *et al.*, 2012). Geriatric nurses, for instance, work an average of 2h per early shift in a forward-bending posture and this proportion rises as residents’ care needs increase (Freitag *et al.*, 2012).

Only a few studies have examined sagittal inclinations in nursing personnel (Hignett, 1996; Caboor *et al.*, 2000; Hodder *et al.*, 2010; Szeto *et al.*, 2013) and most of those focused on patient transfers and paid scant attention to other care activities. As yet, it is an unanswered question how to reduce the large number of inclinations for nursing personnel. Whether nursing personnel perceive a reduction in bending as a physical relief also has yet to be established.

Therefore, we conducted a laboratory study to ascertain whether the following two factors affect the proportion of time nursing personnel spend working in a forward-bending posture: (i) the height of the bed during basic care activities at the bedside and (ii) the work method (standing, kneeling, and sitting) during basic care activities in the bathroom. We also investigated the connection between the proportion of time spent in a forward-bending posture and the perceived exertion.

## METHODS

### Study population

A convenience sample of 12 nurses agreed to participate in the study (Table 1). The nurses had to be free from back complaints when the measurements were taken (inclusion criterion)—that means, nurses who were unable to perform certain kinds of work due to acute back pain were excluded from participating in the study.

**Table 1.** Description of study population (***n*** = 12)

Characteristics	Values
Age (years)	
<30	4 (33.3%)
30–40	5 (41.7%)
>45	3 (25.0%)
Height (cm)	166.1 (±6.6)
BMI (kg/m^2^)	23.4 (±3.3)
Professional experience (years)	7.6 (±7.1)
Gender (female)	10 (83.3%)
Registered nurse	6 (50.0%)

BMI = body mass index. Metric values are given as mean values (standard deviation). Category values are given in numbers (percentages).

### Measurement procedure

The investigations were conducted in the geriatric nursing home where all 12 participants worked. The nursing home placed one room with two care places and two identical bathrooms at our disposal. Two colleagues in our research group volunteered as patients. They were instructed neither to help the care givers nor to resist the care activities. Each participant performed a standardized routine of different care activities at the bedside and then in the bathroom. Bedside care activities included washing and drying the arms, legs, feet and back of the patient, and changing the bed sheet. All participants learned the standardized routine and performed two test runs. Next, each participant performed the care routine three times in succession at three different bed heights (Table 2): (i) with the upper edge of the mattress at knee height, (ii) at mid-thigh height, and (iii) at hip height (Fig. 1). In each case, the bed heights were geared to the height of the individual test person. Immediately after each test, the participants rated how physically strenuous they perceived performing the care activities at the particular bed height to be. For the assessment, we used the 15-point Borg’s RPE scale for rating (R) perceived (P) exertion (E). Scale values ranged from 6 (no exertion at all) to 20 (exhaustion) (Table 3) (Borg, 1990).

**Table 2.** Duration of measurements and proportion of trunk inclination in different angle classes, separated by setting

Setting	Duration of measurements	Proportion of inclination, M ± SD (%)			
	M ± SD (min)	<20°	20°–40°	40°–60°	>60°
Bed setting					
Knee height	7.45±0.92	18.5±4.6	19.5±7.1	33.6±7.2	28.4±13.6
Thigh height	7.79±1.24	26.4±8.7	34.6±10.7	32.8±10.8	6.3±8.7
Hip height	8.47±1.66	45.4±15.5	42.5±8.3	11.8±8.8	0.2±0.3
Bathroom setting					
Standing	2.44±0.53	13.1±4.9	7.9±3.8	6.3±5.2	72.7±10.7
Kneeling	2.27±0.74	32.7±30.4	44.7±20.7	20.1±17.7	2.5±3.5
Sitting	2.38±0.42	38.9±23.7	27.5±12.8	28.3±19.2	5.3±6.9

M ± SD = mean ± standard deviation.

**Table 3.** Perceived exertion measured by Borg scale, separated by setting

Setting	Perceived exertion, M ± SD (points^a^)
Bed setting	
Knee height	17.3±1.5
Thigh height	12.6±2.7
Hip height	9.8±2.0
Bathroom setting	
Standing	17.2±1.7
Kneeling	11.0±2.0
Sitting	9.7±2.5

M ± SD = mean ± standard deviation.

^a^Borg scale rating; 6 = no exertion at all and 20 = maximum exertion.

**1:**
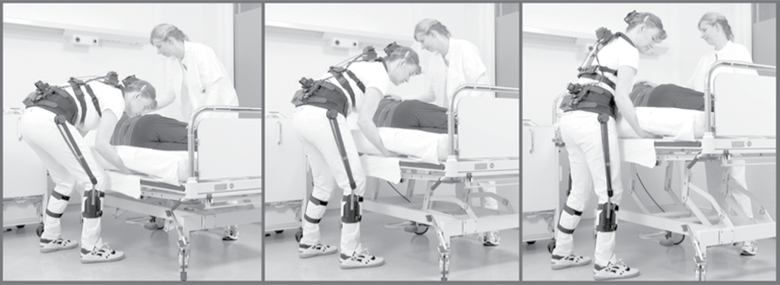
Standardized routine of different care activities at three different bed heights: upper edge of mattress at knee height, thigh height, and hip height.

After the bedside tests, the bathroom tests were conducted. The participants performed a standardized care routine, with the patient sitting on a chair at the washbasin. The bathroom care activities included the washing and drying of legs and feet, and putting on pantyhose. The participants performed the standardized routine in the bathroom three times in succession (Table 2), adopting three different work methods: (i) standing, (ii) kneeling or squatting, and (iii) sitting on a stool (Fig. 2). Immediately after each test, the participants rated on the Borg scale how physically strenuous they perceived the work routine to be when adopting the particular work method.

**2:**
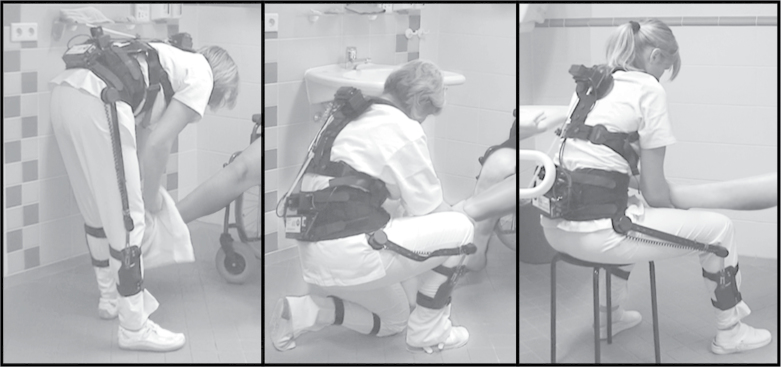
Standardized routine of different care activities in the bathroom adopting three different work methods: standing, kneeling or squatting, and sitting on a stool.

### The CUELA measurement system

The mean trunk inclination in the sagittal direction (hereinafter referred to as ‘inclination’) was measured using the CUELA (German abbreviation for ‘computer-assisted recording and long-term analysis of musculoskeletal loads’) measurement system (Ellegast *et al.*, 2009) (Figs 1 and 2). Sensors at the thoracic and lumbar spine were sampled at a frequency of 50 Hz, so we were able to capture a realistic record of the nurses’ movements. All necessary system components (weighing ~2.7kg) were attached to the participants’ bodies over their work clothing. Adjustable elastic belts were used to adapt the system to the individual participants’ body shapes and to ensure that system components did not slip out of place. Since all system components were carried on the person, no connections to external components were required and the participants could move freely during the measurement process. In addition, we recorded all measurements with a video camera. After completing the measurements, we used specially developed WIDAAN 2.79 software to synchronize the video recordings with the measurement data. As a result, we were able to match each body posture recorded with the corresponding work situation.

### Evaluation of trunk postures

According to the International Organization for Standardisation (ISO) 11226 and DIN EN 1005-4 standards (ISO, 2000; DIN, 2005), trunk inclinations from 0° to 20° are defined as acceptable and correspond to an upright trunk posture. Pressure on the vertebral discs is lowest in this position and climbs as the degree of inclination increases (Wilke *et al.*, 1999). We therefore examined the proportions of time that the participants spent in the following classes of angle of inclination: 0°–20°, 20°–40°, 40°–60°, and >60°.

### Statistical analyses

Prior to analysis, distribution of data was graphically checked and, if necessary, transformed by taking the logarithm. Categorical data are presented as count (percentages), and continuous data are expressed as mean with standard deviations. Comparisons of trunk inclination and Borg scale data among the different working methods were performed by mixed models with nurses as random effects, adjusted for age, gender, and duration of measurement. Interaction between age and gender was checked, but *P* values were not significant for all variables investigated. We report marginal means with 95% confidence limits. *P* values were reported without correction for multiple testing. *P* values <0.05, two sided, are considered statistically significant. Statistical analysis was performed using the SPSS statistical software package 20 (IBM SPSS Statistics Inc., Chicago, IL, USA).

## RESULTS

All 12 participants performed three tests at the bedside and three in the bathroom, so a total of 72 tests were conducted: 36 at the bedside and 36 in the bathroom. All participants said that, after putting on the measurement system, it took a little while for them to get used to it, but after the two test runs, the system was no problem to wear and did not interfere with their performance of care activities.

### Bedside tests

When the bed was at knee height, the participants spent 18.5% (±4.6) of the test duration in an upright position and 81.5% (±4.6) bending forward at an angle of >20° (Table 2). The proportion of very pronounced inclinations of >60° was 28.4% (±13.4). When the bed was set at thigh height, the proportion of time spent in an upright position increased by 7.9–26.4% points (±8.7) and the proportion of very pronounced inclinations of >60° decreased by 22.1–6.3% points (±8.7). With the bed at hip height, there were almost no inclinations of >60° and the participants spent almost half the time working in an upright position.

With all three measurements recorded at the bedside, all participants gave the highest Borg rating to work at the lowest bed setting (knee height) with a mean of 17.3 (±1.5), corresponding to the ‘very hard’ rating on the Borg scale (Table 3). When the bed was set to thigh level, all participants gave a lower rating than on the knee-high setting with a mean of 12.6 (±2.7). This rating corresponds to the perceived exertion ‘somewhat hard’. The highest bed setting, at hip height, received an average rating of 9.8 (±2.0), which corresponds to an exertion between ‘very light’ and ‘light’.

### Bathroom tests

When the work method adopted was ‘standing’, the participants spent 13.1% (±4.9) of the test duration in an upright position and 86.9% (±4.9) bending forward at an angle of >20° (Table 2). The proportion of very pronounced inclinations of >60° was 72.7% (±10.7). When the participants worked in a kneeling or squatting position, the proportion of time spent in an upright position increased by 19.6–32.7% points (±30.4) and the proportion of inclinations of >60° fell considerably by 70.2–2.5% points (±3.5). When the participants worked sitting on a stool, the proportion of time spent in an upright position rose by an additional 6.2% points, but the proportion of very pronounced inclinations of >60° rose by 3.5% points. The subjects’ work method varied widely when they used a stool. Some laid the leg of the patient across their own thigh, while others just lifted it slightly or left it on the floor. Four participants placed the patient’s leg on their thigh and consequently spent two and a half times the percentage of time in an upright position than the other participants [62.5% (±22.6) versus 27.1% (±13.8); data not shown].

With all three measurements recorded in the bathroom, all participants awarded the highest Borg rating to the ‘standing’ work method with a mean of 17.2 (±1.7) (Table 3). This corresponds to the exertion ‘very hard’ on the Borg scale. All participants awarded a lower rating to the ‘kneeling’ work method, on average 11.0 (±2.1), than to the ‘standing’ work method. This corresponds to the perception ‘light’. On average, the work method ‘sitting on a stool’ was given the lowest Borg rating with a mean of 9.7 (±2.6). This corresponds to an exertion between ‘very light’ and ‘light’. When one compares the participants who placed the patient’s leg on their thigh while sitting on the stool with the other participants, the former group gave a mean Borg rating of 7.5 (±0.6) and the latter a rating of 10.8 (±2.6) (data not shown).

Both the bed height and the work method in the bathroom influenced the proportion of time spent in an upright trunk posture (Table 4). When the bed was moved from knee to thigh height, the proportion of time in an upright position increased by 8.2% points, but the effect was not significant (*P* = 0.193). When the bed was moved to hip height, there was a significant increase of 19.8% points (reference: thigh height; *P* = 0.003) and 28.0% points (reference: knee height; *P* < 0.001). In the bathroom setting, we found a similar situation: compared to the standing work method, the kneeling and sitting work methods led to a significant increase in the proportion of time spent upright, of 19.4% points (*P* = 0.003) and 25.7% points (*P* < 0.001), respectively. The model was adjusted for age, gender, and duration of measurement, but these variables showed no significant effect. The intra-class correlation (ICC) concerning proportion of time in an upright posture was 0.25 (*P* = 0.121) and the ICC concerning perceived exertion was 0.004 (*P* = 0.959).

**Table 4.** Parameter estimates of fixed effects for the primary outcome variable ‘proportion of time in an upright posture’

Parameter	Estimate	95% Confidence interval		*P* value
		Lower	Upper	
Intercept	37.2	8.4	65.9	0.015
Gender				
Female	−6.3	−26.6	14.0	0.482
Male	0^a^			
Duration (min)	−1.0	−5.7	3.6	0.661
Setting				
Bed: knee height	−15.2	−41.8	11.4	0.257
Bed: thigh height	−7.0	−35.0	21.0	0.618
Bed: hip height	12.8	−18.1	43.6	0.412
Bathroom: standing	−25.7	−38.1	−13.3	<0.001
Bathroom: kneeling	−6.4	−18.8	6.0	0.308
Bathroom: sitting	0^a^			
Age (years)				
<30	14.4	−5.4	34.2	0.127
30–45	11.1	−7.7	29.9	0.201
>45	0^a^			

^a^Reference category (parameter set to zero).

A greater proportion of time spent in a forward-bending posture led to an increased perception of physical exertion (Table 5): an increase of 10% points in the proportion of inclination over 20° increased the Borg rating significantly, by 1.2 points. Age also had a significant effect when comparing the youngest (<30 years) and oldest (>45 years) participant groups. The younger participants rated their perceived exertion at 2.2 points higher on the Borg scale. Adjustment for gender and duration of measurement had no significant effect.

**Table 5.** Parameter estimates of fixed effects for the primary outcome variable ‘perceived exertion’ (Borg rating)

Parameter	Estimate	95% Confidence interval		*P* value
		Lower	Upper	
Intercept	1.65	−2.63	5.93	0.444
Gender				
Female	1.83	−0.23	3.89	0.080
Male	0^a^			
Duration (min)	0.08	−0.17	0.33	0.538
Age (years)				
<30	2.16	0.08	4.25	0.042
30–45	0.77	−1.18	2.73	0.432
>45	0^a^			
Inclination^b^ > 20°	0.12	0.08	0.16	<0.001

^a^Reference category (parameter set to zero).

^b^Inclination = proportion of time in a forward bending trunk posture.

## DISCUSSION

We found that as bed height increases, participants spend longer working in an upright position. However, we also found that although raising the bed from knee to thigh level reduced very pronounced inclinations (>60°) considerably, there was only a slight improvement in the proportion of time spent in an upright position. Only when the bed was raised to hip height, the participants spent a significantly greater proportion of time in an upright posture. We also found that the greater the proportion of time the participants spent in an upright position, the lower their perceived exertion was. This association was confirmed in the bathroom tests: the participants spent the largest proportion of time in an upright position when a stool was used and perceived this work method as requiring the least exertion.

The results show that ergonomic posture can help to reduce physical strain considerably. The degree of potential relief becomes clear in connection with the results of our previous study (Freitag *et al.*, 2012): we found that the nursing personnel spent an average of 2h (geriatric care) and 1h (nursing) in a forward-bending position on the morning shift. This proportion of time rose significantly when more basic care activities were performed. In the previous study, we also observed that none of the 27 nursing personnel adjusted the bed to hip height when performing basic care, even though there were height-adjustable beds in many of the test wards.

Our results are in accordance with other authors who showed that adjusting the bed height leads to more upright trunk posture. Additionally, these authors showed that more upright trunk posture results in lower compressive and shearing force in the lumbar spine (Gagnon *et al.*, 1987; de Looze *et al.*, 1994; Caboor *et al.*, 2000). However, these studies examined only transfer activities. Caboor and de Looze also specified standard bed heights of 51.5 and 71.5cm, regardless of the height of individual participants. The participants were then given a free choice of bed height, but both authors ascertained that only a few of the participants altered the bed height significantly, by a mean value of +6.4 and +4.0cm, respectively. Thus, both authors concluded that most care staff do not know which bed height is best for them.

A further reason for the failure to adjust beds to hip height might be a lack of awareness on the part of nursing personnel of how often they incline their trunk forward. Only our previous studies delivered specific figures on this (Freitag *et al.*, 2007, 2012). In addition, nursing personnel think that adjusting the height of beds takes too much time (Petzäll *et al.*, 2001). This is a misapprehension, however, given that modern electric nursing beds (including a 75kg load) can be adjusted by ~1.5cm s^−1^ on average (according to information supplied by seven German bed manufacturers). Accordingly, it takes 53 or 59 s, respectively, to raise a bed from knee to hip height, assuming an average height difference of ~35cm for women and 39cm for men (SizeGERMANY, 2009). Geriatric nurses perform an average of 6.2 basic care routines per early shift, and hospital nurses an average of 3.4 basic care routines (Freitag *et al.*, 2012). If the beds were to be raised from knee to hip height for each basic care routine, e.g. female nursing staff would have to invest a total of only 5.5 or 3.0min per early shift in adjusting bed heights.

Also Caboor *et al.* (2000) used the 15-point Borg scale to examine whether adjusting the bed height influences perceived exertion. However, the research group found no difference in perceived exertion (12.85 as opposed to 12.93). The authors assumed that the small difference in bed heights (a mean of +6.4 and +4.0cm) had been too minor to have any effect. Our results support this assumption, as the clear differences between our three bed heights had a significant effect on perceived exertion.

A questionnaire-based investigation by Trinkoff *et al.* (2003) appears to argue against the use of height-adjustable beds. The research group came to the conclusion that the presence of height-adjustable beds was associated with a greater likelihood of back complaints, among other things. This might be true, but it is not height-adjustable beds that should be seen as the cause of back complaints. It is rather the fact that this type of bed is most often used for patients in high need of care (Freitag *et al.*, 2007, 2012).

Several limitations should be pointed out: the participants were not selected at random but as a convenience sample, whereby all the participants worked in the same nursing home. This could make the results less generalizable, but we assume that the population bias is slight since the institution is a typical German nursing home in which the care routines under investigation form part of the daily routine. Additionally, the individual nurse explains only 25% of the variance for trunk inclination and for the perceived exertion even <1%. Most participants were unaccustomed to working with the bed at hip height and were rather reluctant to use a stool in the bathroom at first. So these work methods, perceived as unaccustomed, may have had an influence on the perceived exertion. We assume that, after a period of habituation, the participants would have rated the new work methods as providing even greater physical relief. The short duration, especially of the care activities in the bathroom, could also have an effect on the rating of perceived exertion—the perceived exertion in the more stressful settings was probably underestimated as a result. Additionally, it is important to point out here that the bed height perceived to be the least strenuous (hip height) only applies to care activities, and not to moving patients, e.g. toward the head of the bed, or out of bed. The conditions that apply in these cases were investigated by Jäger *et al*. (2013).

Our results show that nursing personnel can play an influential role in designing their work more ergonomically. For the provision of basic care to patients or residents, it is worthwhile to adjust the bed consistently to hip height. The greater the care needs, the more stressful postures can be avoided by doing so. For basic care in the bathroom, especially when dealing with patients’ legs and feet, a stool should be used. As a result, nurses neither have to bend down low from a standing position nor kneel on the floor in front of the patient, which can be problematic, especially for older nursing personnel. Moreover, this has a positive side effect: if the nurse is sitting on a stool, he or she is at eye and ear level with the patient or resident. This leads to improved communication possibilities, especially with older people, whose vision and hearing is often limited.

## CONCLUSIONS

In summary, the present study shows that there is a significant connection between the work method, the proportion of time in an upright posture and perceived exertion, when performing selected basic care activities at the bedside or in the bathroom. Raising the bed to hip height and using a stool in the bathroom significantly increases the proportion of time that nursing personnel work in an upright position. These work methods are perceived as requiring the least exertion.

Around 5min invested in raising beds to hip height will allow nurses to work in an ergonomic posture for most of their shift. Reduction in these risks for back injury requires both institutional provision of enabling furniture and the effective use of these devices by nursing staff.

## FUNDING

No funding was received.
